# Does Counting Different Life Stages Impact Estimates for Extinction Probabilities for Tsetse (*Glossina* spp)?

**DOI:** 10.1007/s11538-021-00924-1

**Published:** 2021-08-02

**Authors:** Elisha B. Are, John W. Hargrove, Jonathan Dushoff

**Affiliations:** 1 Centre of Excellence in Epidemiological Modelling and Analysis (SACEMA), University of Stellenbosch, Stellenbosch, South Africa; 2 Department of Mathematics, Simon Fraser University, Burnaby, BC Canada; 3 Department of Biology, McMaster University, Hamilton, ON Canada

**Keywords:** Extinction probability, Insect population dynamics, Tsetse (*Glossina* spp), Geometric distribution, 60D05, 92B05, 60G50

## Abstract

As insect populations decline, due to climate change and other environmental disruptions, there has been an increased interest in understanding extinction probabilities. Generally, the life cycle of insects occurs in well-defined stages: when counting insects, questions naturally arise about which life stage to count. Using tsetse flies (vectors of trypanosomiasis) as a case study, we develop a model that works when different life stages are counted. Previous branching process models for tsetse populations only explicitly represent newly emerged adult female tsetse and use that subpopulation to keep track of population growth/decline. Here, we directly model other life stages. We analyse reproduction numbers and extinction probabilities and show that several previous models used for estimating extinction probabilities for tsetse populations are special cases of the current model. We confirm that the reproduction number is the same regardless of which life stage is counted, and show how the extinction probability depends on which life stage we start from. We demonstrate, and provide a biological explanation for, a simple relationship between extinction probabilities for the different life stages, based on the probability of recruitment between stages. These results offer insights into insect population dynamics and provide tools that will help with more detailed models of tsetse populations. Population dynamics studies of insects should be clear about life stages and counting points.

## Introduction

Insects play key ecological roles, both positive and negative, for the health of plants and animals, including humans, and for the environment in general (Ollerton et al. 2011; Öckinger and Smith 2007). Many are important vectors of plant and animal diseases, often of public health importance (Tobias 2016; Wamwiri and Changasi 2016; Beier 1998); others are beneficial in, for example, pollination, and some serve as a source of protein for massive numbers of species of animals including humans (Ramos-Elorduy et al. 1997). Biologists are accordingly interested in insect population persistence for various reasons. Conservationists are concerned about the ecological implications of extinction of insect populations, while vector biologists are interested in controlling or eliminating insect vectors of disease (Burt 2014; Shaw et al. 2013; Hocking et al. 1963).

There is evidence of steep declines in insect populations in different parts of the world (Conrad et al. 2002; Potts et al. 2010; Ilyinykh 2011; van Swaay et al. 2013; Lister and Garcia 2018). Hallmann et al. (2017) reported a decline of 75% in the biomass of flying insects over a 27-year period in 63 protected areas of Germany. Similar findings of major decline have been reported across the globe (Habel et al. 2016; Pelton et al. 2019). For instance, there was a decline of 50% in the population abundance of European grassland butterflies between 1990 and 2011 (van Swaay et al. 2013), and it has been reported that tsetse populations have been declining in the Zambezi Valley of Zimbabwe (Lord et al. 2018). If the magnitude of the declines is as serious as reported, the earth may soon witness extinction of large numbers of insect species.

Insects have limited thermo-regulatory capacity, making them particularly vulnerable to changing temperature regimes—in particular, to the effects of global climate change. There is therefore a growing interest in how increases in global temperature will impact insect populations. Questions about extinction of insect populations are now being asked more frequently (Nilsson et al. 2008). Accordingly, there is a need to continue to improve the accuracy of our prediction of the probability of extinction events in insects—and indeed other animals and plants.

The life history of insects occurs in well-defined stages. The question thus arises as to how the developmental stage of counted individuals affects demographic conclusions—for example, the probability that a population will go extinct. We investigate this problem, using populations of tsetse (*Glossina* spp.) as an example. Tsetse are vectors of trypanosomiasis (Wamwiri and Changasi 2016; Kioy et al. 2004), a deadly disease called African sleeping sickness in humans and *nagana* in livestock (Kioy et al. 2004). The life cycle of the fly involves five distinct stages, namely egg, larva, pupa, newly emerged adult, and mature adult (Ackley and Hargrove 2017). Here, for simplicity, we considered three distinct tsetse life stages—particularly since the other two stages (egg and larval) are all completed within the adult mother’s uterus. We ask: how would counting different insect life stages affect our calculation of the probability that an insect population will persist under various circumstances?

Several researchers have developed mathematical models to explore different phenomena in insect population dynamics, but are generally not clear about which developmental stage(s) are being counted (Ylioja et al. 1999; Artzrouni and Gouteux 2003; Hargrove 2005; Adams et al. 2005; Barclay and Vreysen 2011; Peck and Bouyer 2012; Lin et al. 2015; Kajunguri et al. 2019). As far as we are aware, no published work has explicitly considered the implication of counting insects at different stages for the estimation of extinction probabilities for insect populations. Hargrove (2005) developed and analysed a branching process model to derive expressions for extinction probabilities, times to extinction, reproduction number and variance for closed populations of tsetse. The results reported were consistent with earlier work (Hargrove 1988) on tsetse vital rates and showed that small increases in adult female mortality rates could drive any closed population of tsetse to extinction. Kajunguri et al. (2019) added proofs and improved on some of the assumptions in Hargrove (2005). Are and Hargrove (2020) extended this work to provide estimates of extinction probabilities, growth rates, reproduction number and times to extinction as a function of ambient temperature.

In the above studies, the modelling framework was built on the assumption that the pioneer population starts with one or more newly emerged adult female tsetse. In the current study, by contrast, we generalize the approach—allowing the pioneer population to be composed of either juveniles, emergent females or mature females. We establish a relationship between the extinction probabilities for tsetse populations where the pioneer population starts at any of these different life stages. We discuss the implications of these results for tsetse population persistence, particularly in the context of tsetse control/eradication exercises.

The analyses here focus specifically on tsetse, but are likely to be applicable to other insects with similar life cycles. The study illuminates general issues involved in considering the life stage distribution across a population for any insect with overlapping generations. The model and analyses we present here provide insights into the understanding of extinction probability estimates for tsetse. These results can be used to improve the understanding of mechanisms that will help improve tsetse control, and inform policy relating to conservation of endangered species with similar life cycles.

### Brief Description of Tsetse Life Cycle

Female tsetse typically mate once in their lifetime: the sperm transferred by a male during mating is sufficient for the female to fertilize all subsequent eggs throughout her life. A female fly produces, typically every 9-11 days, a single larva, which may weigh as much or even more than she does herself. The larva burrows into the soil and pupates within minutes. The pupal period lasts 30–50 days (Phelps and Burrows 1969), depending on soil temperature. After the pupal period, an immature adult emerges. It takes 7–9 days, depending on temperature, for the newly emerged adults to attain full maturation. During this period, females are typically inseminated by a male tsetse, and virtually all will have ovulated by the age of 10 days (Hargrove 2012). The fully developed adult typically larviposits every 9–11 days afterwards, again depending on temperature (Hargrove et al. 2019).

## Mathematical Model

**Fig. 1:**
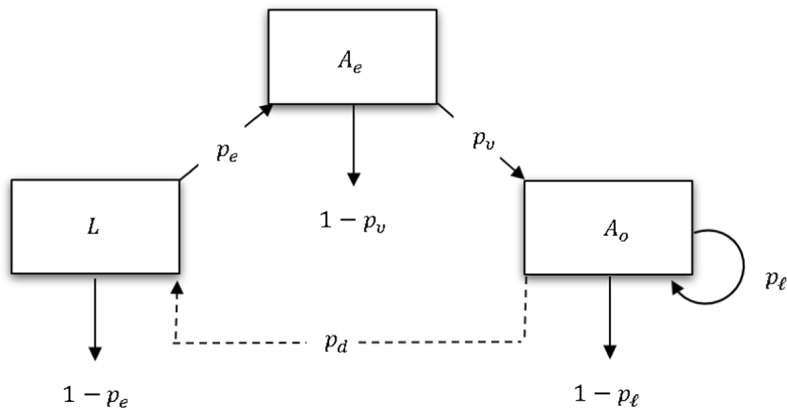
Schematic diagram for tsetse life cycle. The directed arrows pointing to, and away from, the boxes indicate various biological processes in the life cycle of a female tsetse. These include larviposition, emergence as young adult, and development from young adult to mature adult. The arrows pointing downward show losses at various life stages. The circular line segment from $A_o$ back onto itself indicates progression around the larviposition loop; $p_d$ gives the probability that a larva is successfully deposited when the loop is transited.

**Fig. 2:**
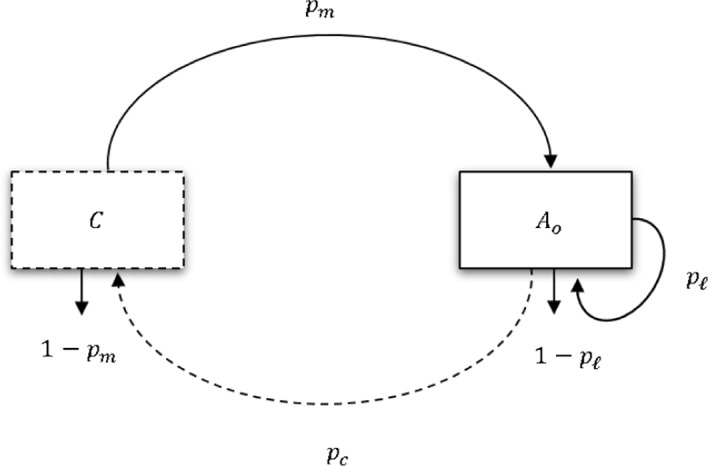
Schematic diagram for counting tsetse. Graphical description of the thinking behind the mathematical formulation of the model. The dashed box around C indicates the life stage at which the individuals are counted (either at the larval, immature adult or mature adult stages). The dashed line from $A_{o}$ to *C* shows the process of producing offspring that are counted, the line is dashed because adults do not become larvae, rather they deposit larvae. And the solid line from *C* to $A_{o}$ indicates the process of being counted, and developing to maturity (reaching the larviposition loop)

Our model of tsetse population dynamics is based on two flow diagrams (Figs. 1 and 2). The first flow diagram illustrates the biological processes associated with the tsetse life cycle. The state variables are described as: *L*, newly deposited larvae, $A_{e}$, emergent adults/newly emerged adults, $A_{o}$, adults in the larviposition loop. The parameters are described as: $p_{\ell }$, probability of completing a larviposition loop, $p_d$, probability of depositing a live female larva, $p_e$, probability a newly deposited female larva emerges as an adult and $p_{\nu }$, probability a newly emerged adult reaches the larviposition loop. Throughout the model formulation and analysis we focus on the female population since a female only needs to mate once in her lifetime, and even with very low numbers of males in the population, females still manage to mate successfully (Glasgow 1963). Moreover, extinction of the female population implies eventual extinction of the entire population, both male and female.

The second flow diagram presents the counting system. This gives a pictorial description of how different life stages of tsetse can be used, as a proxy, to estimate extinction probabilities for the population. The dashed box shows the life stage chosen for counting, which can either be newly deposited larvae, newly emerged adults or larvipositing adults. The *C* inside the dashed box indicates the point where tsetse are counted, while $A_{o}$ is as described above. The counting point can vary: one may choose to count tsetse at the juvenile stages, or at the mature stages, i.e. larvipositing females. The framework we present here allows us to calculate the extinction probability and the basic reproduction number for a tsetse population, for all possible counting stages.

For the computational flow diagram (Fig. 2), we used the following state variables and parameter descriptions for the analysis. This is to provide a general result for counting different life stages of tsetse.

- $A_{o}$: Adults in the larviposition loop
- *C*: The census point
- $p_m$: Probability of surviving from being counted to becoming “mature” (entering the loop)
- $p_c$: Probability of surviving from when the larva is deposited after loop completion to becoming something that is counted.
- $p_r$: Probability of recruitment into the larviposition loop ($p_r = p_mp_c$)

### Model Formulation

To make our mathematical derivations simple and compact, we use the *odds* associated with the probability that a female completes a larviposition loop and the probability that it produces a female offspring before dying, to derive the offspring distribution function for tsetse populations. We assume that an individual at the census point (the life stage we choose to count): (i) reaches the loop with probability $p_m$ and (ii) either goes around the loop producing offspring that will be counted, or else dies in the process.

The probability $p_b$ of producing before dying has odds of:

1 $$\begin{aligned} \sigma _b = \frac{p_b}{1-p_b} = \frac{p_\ell p_c}{1-p_\ell } = \sigma _\ell p_c \end{aligned}$$

$p_b$ is the probability of an $A_o$ producing a countable offspring before dying. The odds of producing a counted offspring before dying is the ratio of the probability of producing ($p_lp_c$) to dying ($1-p_l$) each time around the loop. The total number of censused individuals produced by a censused individual is 0, if it does not reach the loop (probability $1-p_m$) and *k*, which could also be 0, if it reaches the loop and then fails after *k* successes, with probability $p_m p_b^k (1-p_b)$.

The associated generating function is:

2 $$\begin{aligned} G(s) = 1-p_m + p_m (1-p_b) \sum _k p_b^k s^k = 1-p_m + \frac{p_m (1-p_b)}{1-p_b s}. \end{aligned}$$

We get $\mathcal{R}$, the reproduction number, by calculating $G'(1)$ (Bartlett 1949), where

3 $$\begin{aligned} G'(s) = \frac{p_m p_b (1-p_b)}{(1-p_b s)^2}. \end{aligned}$$

Hence,

4 $$\begin{aligned} \mathcal{R}= G'(1) = \frac{p_m p_b}{(1-p_b)} = p_m \sigma _b = p_mp_c \sigma _\ell = p_r\sigma _\ell . \end{aligned}$$

The recruitment probabilities $p_m$ and $p_c$ depend on the counting point, but their product $p_r$ will remain the same. Therefore, $\mathcal{R} =p_dp_ep_{\nu } \sigma _{\ell }$. In other words, the reproduction number is independent of the counting point: the expected number of larvae produced by a larva is equal to the expected number of emergent adults produced by an emergent adult, and so forth.

We get the probability *y* that a tsetse population, starting with a single individual at a given count point, goes extinct, by solving $G(y) = y$ (Bartlett 1949). We can simplify the calculation by solving $G(1-z) = 1-z$, where *z* is the probability of not going extinct, and then factoring out a *z*. This gives:

$$\begin{aligned} z = p_m(1-1/\mathcal{R}). \end{aligned}$$

By factoring out the *z*, we are assuming that the process is supercritical, that is, $\mathcal{R} > 1$. When $\mathcal{R} \le 1$, the population will eventually go extinct with probability 1.

Thus, the probability of extinction for tsetse populations at any (supercritical) counting point is:

5 $$\begin{aligned} y = 1 - p_m(1-1/\mathcal{R}). \end{aligned}$$

When there is more than one individual in the pioneer population, we calculate the extinction probability by assuming that density dependence is negligible, and that overall extinction would therefore result from the independent extinction of the line starting from each individual. Thus, the extinction probability is:

6 $$\begin{aligned} y_j = (1 - p_m(1-1/\mathcal{R}))^j, \end{aligned}$$

where *j* is the number of individuals in the given stage. We can derive extinction probabilities for tsetse populations at the individual counting points by making simple substitutions for the probabilities of recruitment between stages in $p_m$ in equation (5), as appropriate.

## Counting Tsetse at Different Life Stages

When we change the counting stages by moving the dashed box (Fig. 2) closer to the ovulation loop, $\mathcal{R}$ stays the same but $p_m$ gets larger until it reaches 1 when the dashed box gets to the ovulation loop. We can thus calculate extinction probabilities for each of the three counting points (larvae, newly emerged, or larvipositing adults). We first ask what happens if we start with a single newly deposited female larva? The larva reaches the larviposition loop (emerges and then matures) with probability $p_m = p_e p_{\nu }$, completes a larvipositing loop with *odds* $\sigma _\ell$, and produces a surviving female larva with probability $p_c = p_d$. When we make appropriate substitutions in *y*, the extinction probability for a population of tsetse with a single newly deposited larva in the initial population is:

7 $$\begin{aligned} y_l = \frac{1-p_{\ell }(1 - p_{d}(1 - p_{e}p_{\nu }))}{p_{\ell }p_{d}}. \end{aligned}$$

This can be written more compactly in terms of $\mathcal{R}$ as:

8 $$\begin{aligned} y_l = 1 - p_ep_{\nu }(1-1/\mathcal{R}). \end{aligned}$$

This corresponds to the situation where we use the sub-population of newly deposited larvae as a proxy for estimating extinction probabilities for a tsetse population. In similar fashion, we can obtain the extinction probability $y_e$ for a tsetse population starting with a single newly emerged adult fly, by substituting $p_m = p_{\nu }$ in *y* above. We find:

9 $$\begin{aligned} y_e=\frac{1- p_{\ell }(1 -p_{d}p_{e}(1- p_{\nu }))}{p_{d}p_{e}p_{\ell }}. \end{aligned}$$

This can also be rewritten in terms of $\mathcal{R}$ as:

10 $$\begin{aligned} y_e = 1 - p_{\nu }(1-1/\mathcal{R}). \end{aligned}$$

Furthermore, when larvipositing females are counted, $p_m$ will be equal to 1. The extinction probability $y_o$ for a population of tsetse starting with a single larvipositing female tsetse in the initial population is therefore:

11 $$\begin{aligned} y_o = \frac{1-p_{\ell }}{p_{d}p_{e}p_{\nu }p_{\ell }}. \end{aligned}$$

This can be expressed in terms of $\mathcal{R}$ as:

12 $$\begin{aligned} y_o = 1/\mathcal{R} \end{aligned}$$

It is easily verifiable that whenever $\mathcal{R}>1$, the following inequality holds:

13 $$\begin{aligned} y_{o}\le y_{e} \le y_{l} \end{aligned}$$

In our analysis so far, we have focused on populations starting with a single individual in the initial population. In the general case, assuming extinction probabilities for the population starting from each individual for each counting points are independent, the extinction probability for a population starting with $N_l$ larvae, $N_e$ newly emerged adult females and $N_o$ larvipositing adult females, is just the product of the individual extinction probabilities.

14 $$\begin{aligned} \tilde{y_c}=y_l^{N_l} y_e^{N_e} y_o^{N_o}. \end{aligned}$$

The current analysis focuses strictly on female tsetse populations. We can account for this by expressing $p_d$ (probability of depositing a live female larva) as: $p_d =\delta \beta$, where $\delta$ is the probability that a deposited larva is alive, and $\beta$ the probability that a deposited larva is female. These two parameters ($\delta$ and $\beta$) will allow us to capture both male/female sex ratio in the population, and the abortion rates in tsetse population. If we set $\beta = 0.5$ and $\delta = 1$, the current model corresponds to the model presented in Hargrove (2005).

Previous estimates for extinction probabilities for tsetse populations are special cases of the current framework. In particular, the models in (Hargrove 2005; Kajunguri et al. 2019; Are and Hargrove 2020) correspond to the scenario presented above—counting newly emerged adults. We can link the previous estimates with the current model by setting $p_{\nu }= \epsilon \Omega ^{\nu }$ , $p_{\ell } =\lambda ^{\tau }$, $p_{d}=\beta$ and $p_{e} = \phi ^{\sigma }$ in *G*(*s*) above, where $\epsilon$ is the probability that a female is inseminated by a fertile male, $\Omega ^{\nu }$, the probability that a newly emerged adult survives until first larviposition, $\lambda ^{\tau }$, the probability that an adult survives until it deposits a pupa (completes a cycle), $\beta$, the probability that a deposited pupa is female, and $\phi ^{\sigma }$, the probability that a deposited pupa emerges. The parameters $\sigma$, $\nu$ and $\tau$ are the duration (in days) of the pupal stage, newly emerged adult stage, and a single larviposition loop, respectively.

## Results and Discussion

Previous estimates of extinction probabilities for tsetse populations use the assumption that the pioneer population is initiated with a number of newly emerged adult female flies, and the probability that such populations go extinct is estimated as a function of different vital rates in the tsetse life cycle. The life cycle of holometabolous insects, such as tsetse, can be divided into five distinct stages, egg, larva, pupa, immature adult, mature (larvipositing) adult-each with distinct physiological features, and with differing responses to various environmental factors. In tsetse, for example, the most vulnerable stage is the newly emerged adult, which also appears to be particularly susceptible to high temperatures (Ackley and Hargrove 2017). Tsetse are unusual in that survival probability is high in the egg and larval stages, which are retained in the mother’s uterus (Hargrove 1999). Here, we have developed a simple, unified model to analyse extinction probabilities when starting from different life stages.

**Fig. 3:**
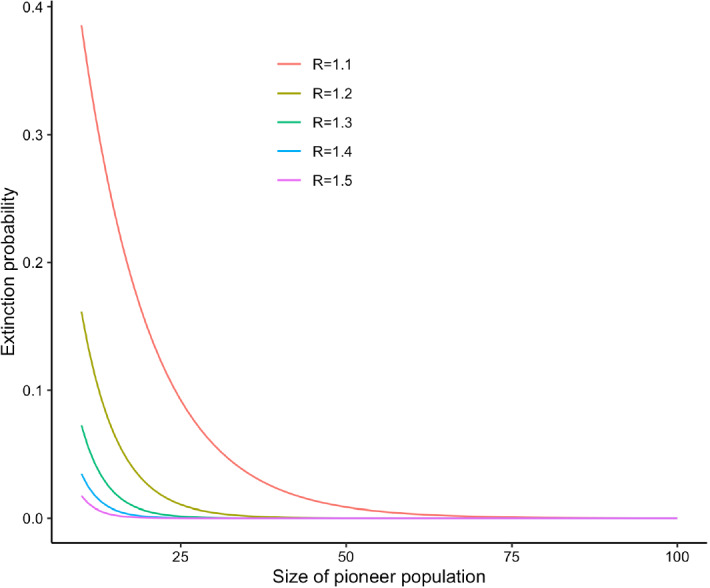
Extinction probability as a function of the size of the pioneer population, for different values of the basic reproduction number. When $\mathcal{R}=1$, the extinction probability is 1 regardless of initial population size

**Fig. 4:**
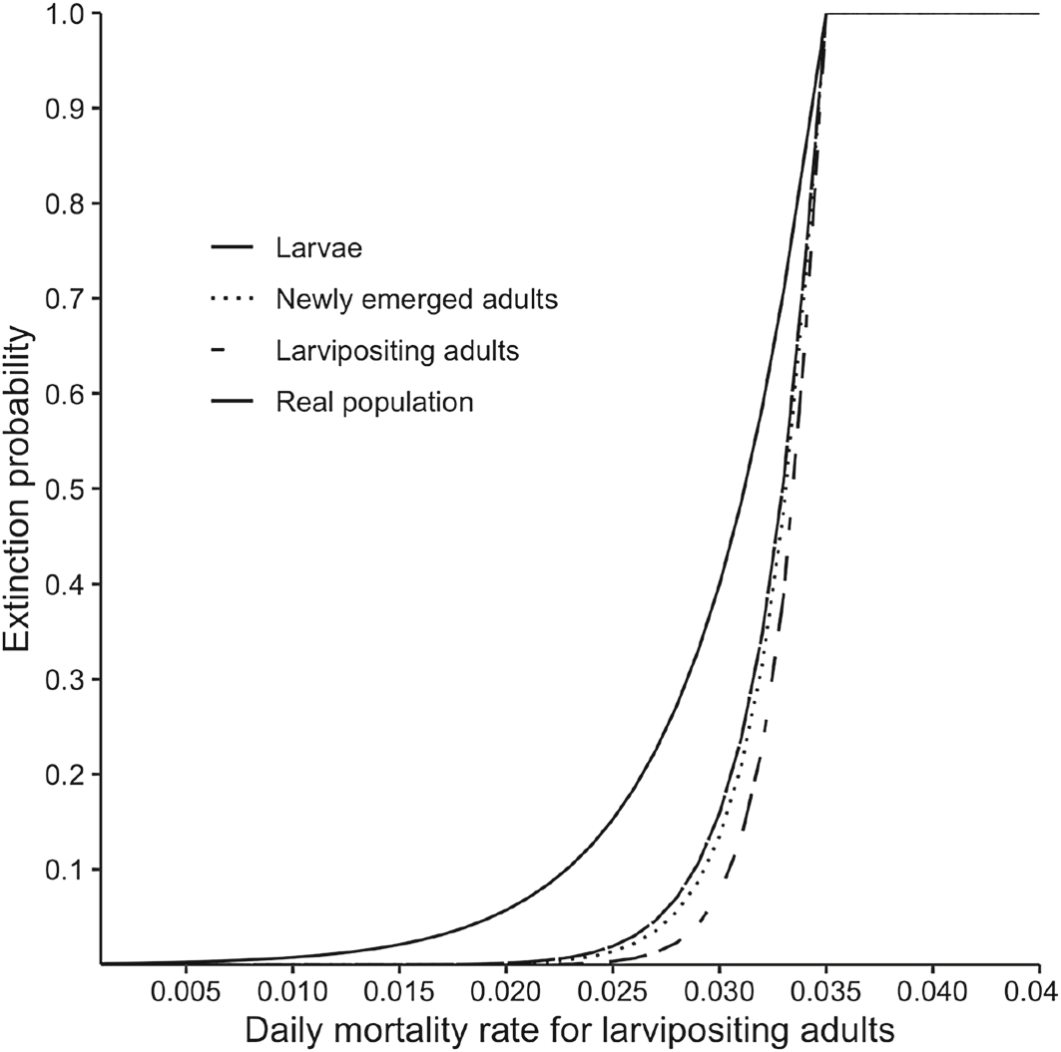
Extinction probability as a function of daily mortality rate for larvipositing adults for different counting systems. Pioneer populations consist of 15 larvipositing or 15 newly emerged adults or 15 larvae or, more realistically, 5 individuals in each of the three life stages

While extinction probabilities change with the life stage of pioneer individuals, decreasing as we move from larvae to mature adults, we have confirmed that the basic reproduction number remains the same for all counting points under a given set of parameters. When the reproduction number is $\mathcal{R} \le 1$, extinction is certain for all the counting systems. Once $\mathcal R$ crosses 1, the extinction probability will fall to zero as $\mathcal R$ increases (Fig. 3). This drop-off gets faster for larger populations: thus, the extinction probability as a function of parameters approaches a step function. This effect can be seen in Fig. 4, where a starting point of 15 larvipositing adults is already stable enough that the extinction probability begins to look like a step function of underlying parameters.

Our modelling framework unifies existing methods for estimating extinction probabilities for tsetse populations. The model only requires a good estimate of the probabilities of recruitment between tsetse life stages to estimate extinction probabilities. Our model presents an opportunity to compare previous estimates for extinction probabilities within a simple unified framework.

When populations are large, extinction probability is either 0 or 1 for all the counting stages. In such situations any life stage can be used as a proxy to estimate extinction probabilities for tsetse populations, as has been done previously (Hargrove 2005; Kajunguri et al. 2019). At low populations, however, counting points become extremely important. For small populations, previous estimates for extinction probabilities underestimated tsetse population persistence when $\mathcal{R} > 1$. But their conclusions are valid for large populations, as well as for the point where extinction becomes certain.

We considered field situations where the population consists of individuals in the three different life stages, and we made a simplifying assumption that the probability of survival and reproduction, in populations starting with any of the life stages, is independent of the individuals in the other life stages. We then obtained the probability that a population which has individuals in all the three life stages goes extinct as the product of the probabilities of extinction for populations starting with only larvae, newly emerged adult or larvipositing adults, respectively.

Figure 4 shows extinction probabilities as a function of the daily mortality rates for larvipositing adults, for situations where the initial population has either only larvae, newly emerged adults, larvipositing females or individuals of all of the life stages. Current results suggest that $\mathcal R$ $<1$ when adult mortality is around 3.5% or higher. Thus, if this level of mortality is sustained, we expect population extinction regardless of initial population size or stage distribution (Fig. 4). This is in good agreement with earlier studies that have suggested the same level of mortality for ensuring eradication of tsetse populations (Hargrove 2005; Kajunguri et al. 2019).

Our model assumes that tsetse populations are not affected by movements between patches. In the field, tsetse populations exist in patches, which means that extinction within a patch does not guarantee extinction of the population, as other neighbouring patches may compensate for the loss through inter-patch migration. In such situations, extinction probabilities are expected to be lower than our estimates here (Peck 2012). Environmental factors such as temperature, humidity, and moisture have a considerable impact on tsetse life cycle. We did not factor in these climatic variables in the current model. However, we expect the principles outlined here to apply to more complex models that incorporate these environmental variables. For simplicity, we assumed that the daily mortality rates remain constant for tsetse in a given life stage. Although studies have shown significant difference between the mortality rates in newly emerged adults and larvipositing adults, once tsetse ovulate for the first time, their mortality rates change very slightly with age, of course depending on temperature (Hargrove 1990). In practice, newly emerged adults and larvipositing adults will be easier to sample than newly deposited larvae, since deposited larvae burrow into the soil in a matter of minutes once deposited (Phelps and Burrows 1969).

The theoretical framework developed previously for estimating extinction probabilities for tsetse populations assumed that the population starts with a finite number of newly emerged adults. We have shown here that the results remain similar when we consider different life stages in the starting population. In particular, if the starting population size is large, the distribution does not matter and survival depends only on the average value of $\mathcal R$.

## Conclusion

The general model works for all counting points, for different decompositions of the recruitment rates between the life stages. We showed that extinction probabilities calculated using different stages for counting depend on the probability of recruitment between these stages. We found that previous models used to estimate extinction probability for tsetse populations are special cases of the general model. Our results suggest that previous methods which used newly emerged adults as a proxy for estimating extinction probabilities give results consistent with the estimates obtained when we considered all life stages. And this is true for both large and small population sizes.

This model generalizes previous models, by unifying the treatment and clarifying the result when the initial population consists of different life stages. We provide a method for allowing counts across the different life stages. We found that, for a large population of tsetse, any of the life stages can be used to estimate the extinction probabilities for tsetse in such situations. When populations are sparse, however, basing the calculations on the number of newly emerged adults gives a more valid estimate of the extinction probability.

We can predict insect population persistence only if we count and calculate carefully, taking account of different stages. We caution that the basic reproduction number is not sufficient to accurately determine insect population persistence. Our results offer insights into population dynamics and provide tools that will help with more detailed models of insect populations. Finally, we advise that population dynamics studies of insects should be clear about life stages and counting points.
